# Supplementary material for: Dissecting endogeneous genetic circuits from first principles
Source: ArXiv. 2024 Jan 29:arXiv:2401.15880v1. Preprint. [Version 1] (PMC10862939)
Supplement: Supplementary file 1 [file NIHPP2401.15880v1-supplement-1.pdf]

Supporting Information for Dissecting endogeneous genetic circuits from first principles

Rosalind Wenshan Pan[1*], Tom Röschinger[1], Kian Faizi[1], Rob Phillips[1,2*]

1 Division of Biology and Biological Engineering, California Institute of Technology, Pasadena, California, United States of America
2 Department of Physics, California Institute of Technology, Pasadena, California, United States of America

* Correspondence: rosalind@caltech.edu and phillips@pboc.caltech.edu

S1 Appendix Definition of probability distributions in the calculation of mutual information

In order to build an information footprint from data, we need to calculate the mutual information between expression levels and the base identity at each position in the sequence, which is defined as

$$I_i = \sum_b \sum_\mu \Pr{}_i(b,\mu) \log_2 \left(\frac{\Pr{}_i(b,\mu)}{\Pr{}_i(b)\Pr(\mu)} \right), \tag{43}$$

where b represents base identity and μ represents expression levels.

As shown in Fig S1(A) and S1(C), we find that binding sites have a higher signal-to-noise ratio in information footprints when the coarse grained approach is taken

$$b = \begin{cases} 0, & \text{if the base is mutated,} \\ 1, & \text{if the base is wild type.} \end{cases} \tag{44}$$

On the other hand, to obtain a distribution of expression levels, sequencing counts are binned into N bins, where the bins are chosen such there is an equal number of sequences in each bin. Again, we observe the highest signal-to-noise ratio for $N = 2$, and see a continuous decrease with increasing number of bins. As shown in Fig S1(B) and S1(C), we observe that signal-to-noise ratio is higher when fewer bins are used to partition expression levels.

These observations may be explained by the fact that increasing the number of states or bins increases the level of noise. When there are more states or more bins, fewer sequences will be present in each bin. This amplifies the hitch-hiking effects discussed in Sec 1.3, leading to a higher level of noise. In addition, since the boundaries between the bins are artificially set, a sequence may be randomly grouped into the N-th bin rather than the adjacent (N-1)-th and (N+1)-th bins simply because the boundaries are set a particular level. This randomness occurs in both the marginal probability distribution for μ and the joint probability distribution, resulting in noise that is increased when more bins are added.

S2 Appendix Analytical calculation of information footprint

To better understand how mutations in the binding sites create signals in the information footprint, we derive the information footprint analytically for a constitutively expressed gene, i.e. the promoter of the gene only has a binding site for RNAP and is not bound by any transcription factors.

Consider a promoter region where the RNAP binding site is l base pairs long and the probability of mutation at each site is θ. Furthermore, the binding energy of RNAP to the wild-type sequence is denoted by $\Delta\varepsilon$ and we assume that at each position within the binding site, a mutation comes with cost $\Delta\Delta\varepsilon$ to the binding energy. Therefore, if there are m mutations in the binding site, the total binding energy between RNAP and the mutant binding site is $\Delta\varepsilon + m\Delta\Delta\varepsilon$.

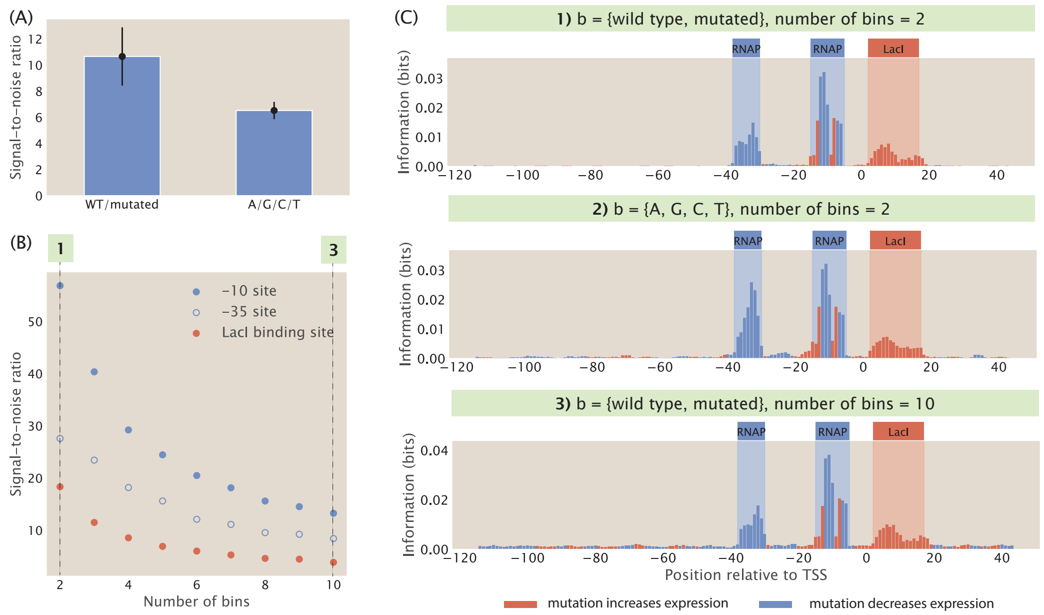

Supplementary Figure 1. Definition of probability distributions in the calculation of mutual information. (A) Using a probability distribution of the four bases leads to a reduced signal-to-noise ratio in the information footprint. The heights of the bars are the average signal-to-noise ratios calculated from the information footprints of 20 synthetic datasets with the repression-activation architecture. (B) Signal-to-noise ratio decreases when the number of bins increases. (C) Choosing different probability distributions to calculate the information footprint for a synthetic dataset with the repression-activation genetic architecture. The top footprint uses a probability distribution of wild-type and mutated bases and uses 2 bins to calculate the probability distribution for expression levels. The middle footprint uses a probability distribution of the four bases (A, G, C, T) and uses 2 bins to calculate the probability distribution for expression levels. The bottom footprint uses a probability distribution of wild-type and mutated bases and uses 10 bins to calculate the probability distribution for expression levels.

In a sufficiently large data set, the ratio of sequences with mutation at position i is given by

$$\mathrm{Pr}_i(b) = \begin{cases} 1-\theta, & \text{if } b = 0 \\ \theta, & \text{if } b = 1. \end{cases} \tag{45}$$

Next, we determine $\mathrm{Pr}(\mu)$. As before, we define $\mathrm{Pr}(\mu)$ as the probability that a given sequence leads to high expression levels or low expression levels. To predict expression levels, we begin by calculating p_{bound} for each promoter variant. Since the gene is constitutively expressed, the probability of RNAP binding is given by

$$p_{\mathrm{bound}} = \frac{\frac{P}{N_{\mathrm{NS}}} e^{-\beta(\Delta\varepsilon + m\Delta\Delta\varepsilon)}}{1 + \frac{P}{N_{\mathrm{NS}}} e^{-\beta(\Delta\varepsilon + m\Delta\Delta\varepsilon)}}. \tag{46}$$

As derived in Eqn 5, the steady state copy number of mRNAs is proportional to the probability of the RNAP bound state. Therefore, expression level is only dependent on the number of mutations in the RNAP binding site.

The probability distribution for the number of mutations in the RNAP binding site can be expressed using the binomial distribution, where the probability of k mutations in the binding site is given by

$$\mathrm{Pr}(m = k; l, \theta) = \binom{l}{k} \theta^k (1-\theta)^{l-k}. \tag{47}$$

As illustrated in Fig S2, ince expression levels are solely determined by the number of mutations in the binding site, and sequences are binned by expression levels to obtain $P(\mu)$, there is a threshold number of mutations, m^*, where sequences with m^* or more than m^* mutations fall into the lower expression bin. Hence, $P(\mu)$ is given by

$$\Pr(\mu) = \begin{cases} \Pr(m \geq m^*; l, \theta) = \sum_{k=m^*}^{l} \binom{l}{k} \theta^k (1-\theta)^{l-k}, & \text{if } \mu = 0 \\ \Pr(m < m^*; l, \theta) = 1 - \Pr(m \geq m^*; l, \theta), & \text{if } \mu = 1. \end{cases} \tag{48}$$

Finally, we determine the expression for $\Pr_i(b, \mu)$. To do this, we consider two cases, one where the position i is outside of the RNAP binding site and one where the position i is within the RNAP binding site. If i is not in the RNAP binding site $\mathcal{B}$, then a mutation would have no effect on the expression levels, therefore

$$\Pr_{i \notin \mathcal{B}}(b, \mu) = \begin{cases} (1-\theta) \cdot \Pr(m \geq m^*; l, \theta), & \text{if } b = 0 \text{ and } \mu = 0 \\ (1-\theta) \cdot \Pr(m < m^*; l, \theta), & \text{if } b = 0 \text{ and } \mu = 1 \\ \theta \cdot \Pr(m \geq m^*; l, \theta), & \text{if } b = 1 \text{ and } \mu = 0 \\ \theta \cdot \Pr(m < m^*; l, \theta), & \text{if } b = 1 \text{ and } \mu = 1. \end{cases} \tag{49}$$

Having derived all the marginal probability distributions and the joint probability distributions, we can then write down mutual information at a non-binding site and at a binding site. If position i is outside the RNAP binding site, then

$$\begin{aligned} I_i =& (1-\theta) \cdot \Pr(m \geq m^*; l, \theta) \log_2 \frac{(1-\theta) \cdot \Pr(m \geq m^*; l, \theta)}{(1-\theta) \cdot \Pr(m \geq m^*; l, \theta)} \\ &+ (1-\theta) \cdot \Pr(m < m^*; l, \theta) \log_2 \frac{(1-\theta) \cdot \Pr(m < m^*; l, \theta)}{(1-\theta) \cdot \Pr(m < m^*; l, \theta)} \\ &+ \theta \cdot \Pr(m \geq m^*; l, \theta) \log_2 \frac{\theta \cdot \Pr(m \geq m^*; l, \theta)}{\theta \cdot \Pr(m \geq m^*; l, \theta)} \\ &+ \theta \cdot \Pr(m < m^*; l, \theta) \log_2 \frac{\theta \cdot \Pr(m < m^*; l, \theta)}{\theta \cdot \Pr(m < m^*; l, \theta)}. \end{aligned} \tag{50}$$

We can see that $I_i = 0$ since the fractions within the logarithms all cancel out to be 1. This is because the joint probability $\Pr_i(b, \mu)$ for bases outside the binding site is simply given by the product of the marginal distributions,

$$\Pr_{i \notin \mathcal{B}}(b, \mu) = \Pr_{i \notin \mathcal{B}}(\mu) \Pr_{i \notin \mathcal{B}}(b). \tag{51}$$

If the position i is in the RNAP binding site, the calculation for $\Pr_i(b, \mu)$ is more complex. As illustrated in Fig S2, if the position i has wild-type base identity, then the sequence would have low expression levels if there are more than m^* mutations in the remaining $l-1$ bases in the RNAP binding site and the sequence would have high expression levels if there are less than m^* mutations in the remaining $l-1$ bases in the RNAP binding site. On the other hand, if the position i is mutated, then the sequence would have low expression levels if there are more than m^*-1 mutations in the remaining $l-1$ bases and the sequence would have high expression levels if there are less than m^*-1 mutations in the remaining $l-1$ bases. Taken together, we can write down the joint probability distribution as

$$\Pr_{i \in \mathcal{B}}(b, \mu) = \begin{cases} (1-\theta) \cdot \Pr(m \geq m^*; l-1, \theta), & \text{if } b = 0 \text{ and } \mu = 0 \\ (1-\theta) \cdot \Pr(m < m^*; l-1, \theta), & \text{if } b = 0 \text{ and } \mu = 1 \\ \theta \cdot \Pr(m \geq m^*-1; l-1, \theta), & \text{if } b = 1 \text{ and } \mu = 0 \\ \theta \cdot \Pr(m < m^*-1; l-1, \theta), & \text{if } b = 1 \text{ and } \mu = 1. \end{cases} \tag{52}$$

In this case, the joint distribution does not factor into the marginal distributions,

$$\Pr_{i \in \mathcal{B}}(b, \mu) \neq \Pr_{i \in \mathcal{B}}(\mu) \Pr_{i \in \mathcal{B}}(b), \tag{53}$$

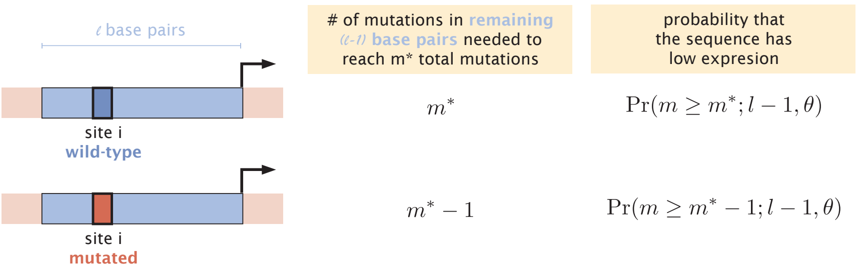

Supplementary Figure 2. Calculating number of mutations needed to reach the threshold between low expression and high expression bins. The joint probability distribution at site i is a product of the probability that site i is mutated or wild type and the probability that the sequence has high or low expression level. Since the expression of the sequence depends on the presence of a mutation at site i, we need to consider two different cases in order to calculate the probability of expression. In the case where site i has wild-type base identity, there need to be m^* mutations outside of site i in the RNAP binding site for the sequence to reach the threshold m^*. Therefore, the probability that the sequence has low expression is $\Pr(m \geq m^*; l-1, \theta)$. On the other hand, in the case where site i is mutated, since one mutation is known to exist, there only need to be $m^* - 1$ mutations outside of site i in the RNAP binding site for the sequence to reach the threshold. In this case, the probability that the sequence has low expression is $\Pr(m \geq m^* - 1; l-1, \theta)$.

and therefore, mutual information has to be larger than zero, $I_i > 0$, clearly distinguishing positions that are within the binding site from positions outside. Specifically,

$$\begin{aligned}
I_i =& (1-\theta) \cdot \Pr(m \geq m^*; l-1, \theta) \log_2 \frac{(1-\theta) \cdot \Pr(m \geq m^*; l-1, \theta)}{(1-\theta) \cdot \Pr(m \geq m^*; l, \theta)} \\
&+ (1-\theta) \cdot \Pr(m < m^*; l-1, \theta) \log_2 \frac{(1-\theta) \cdot \Pr(m < m^*; l-1, \theta)}{(1-\theta) \cdot \Pr(m < m^*; l, \theta)} \\
&+ \theta \cdot \Pr(m \geq m^* - 1; l-1, \theta) \log_2 \frac{\theta \cdot \Pr(m \geq m^* - 1; l-1, \theta)}{\theta \cdot \Pr(m \geq m^*; l, \theta)} \\
&+ \theta \cdot \Pr(m < m^* - 1; l-1, \theta) \log_2 \frac{\theta \cdot \Pr(m < m^* - 1; l-1, \theta)}{\theta \cdot \Pr(m < m^*; l, \theta)} \\
=& (1-\theta) \cdot \Pr(m \geq m^*; l-1, \theta) \log_2 \frac{\Pr(m \geq m^*; l-1, \theta)}{\Pr(m \geq m^*; l, \theta)} \\
&+ (1-\theta) \cdot \Pr(m < m^*; l-1, \theta) \log_2 \frac{\Pr(m < m^*; l-1, \theta)}{\Pr(m < m^*; l, \theta)} \\
&+ \theta \cdot \Pr(m \geq m^* - 1; l-1, \theta) \log_2 \frac{\Pr(m \geq m^* - 1; l-1, \theta)}{\Pr(m \geq m^*; l, \theta)} \\
&+ \theta \cdot \Pr(m < m^* - 1; l-1, \theta) \log_2 \frac{\Pr(m < m^* - 1; l-1, \theta)}{\Pr(m < m^*; l, \theta)}.
\end{aligned} \tag{54}$$

S3 Appendix States-and-weights models for common regulatory architectures

There are six common regulatory architectures for promoters in *E. coli*. Based on the states-and-weights diagrams shown in Fig S3, we can write down p_{bound}, the probability that the RNAP is bound to the promoter, for each of the regulatory architectures [1].

For a constitutively expressed promoter, the states-and-weights diagram is shown in Fig S3(A), and the

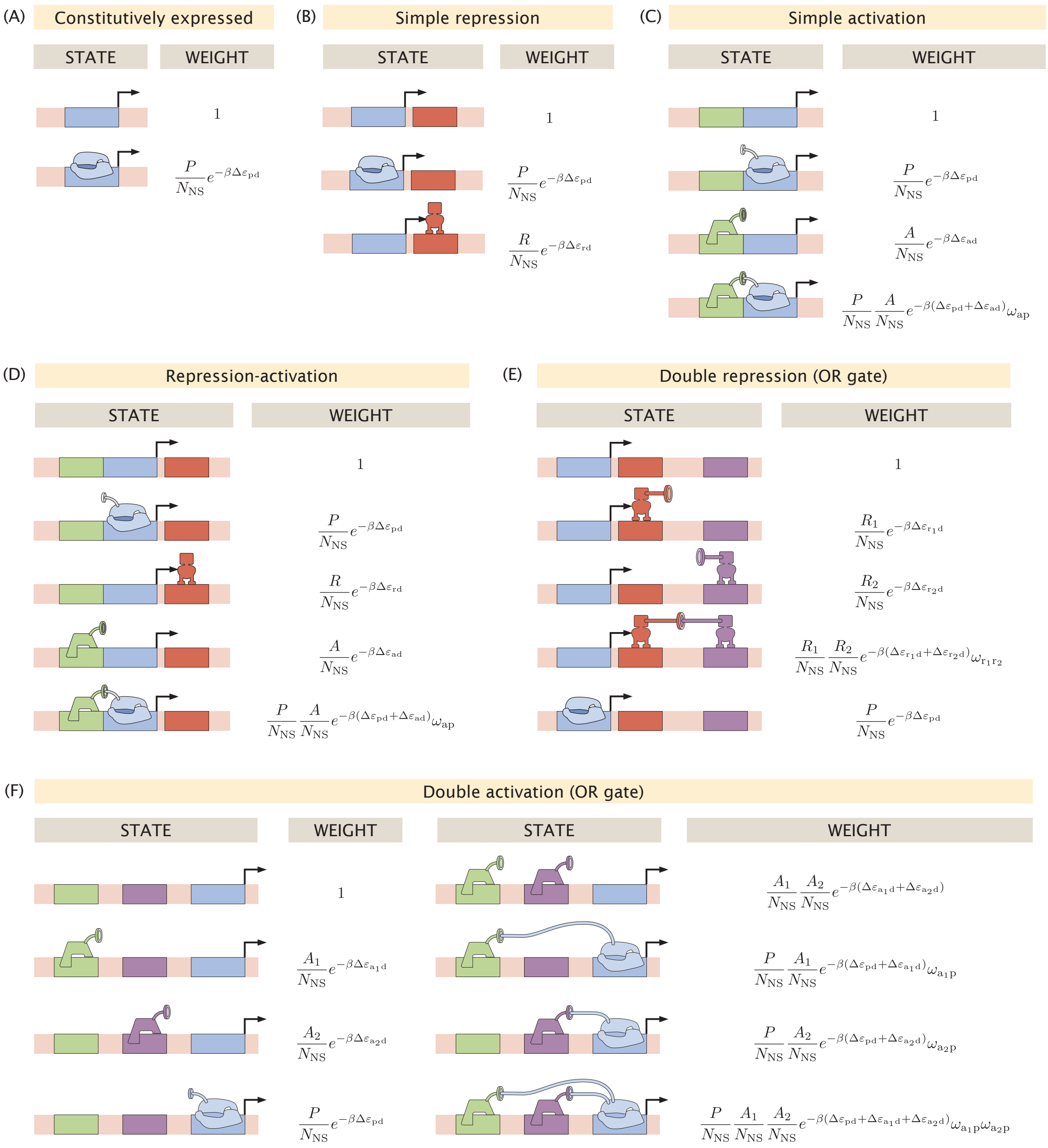

Supplementary Figure 3. States-and-weights models for common regulatory architectures. In all the diagrams, P represents the number of RNAP; R represents the number of repressors; A represents the number of activators; N_{NS} represents the number of non-specific binding sites; $\Delta\varepsilon_{\mathrm{pd}}$ represents the binding energy of the RNAP; $\Delta\varepsilon_{\mathrm{rd}}$ represents the binding energy of the repressor; $\Delta\varepsilon_{\mathrm{ad}}$ represents the binding energy of the activator; ω_{ab} represents the interaction energy between a and b. (A) States-and-weights model for a promoter that is constitutively expressed. (B) States-and-weights model for a promoter under the simple repression regulatory architecture. (C) States-and-weights model for a promoter under the simple activation regulatory architecture. (D) States-and-weights model for a promoter under the repression-activation regulatory architecture. (E) States-and-weights model for a promoter under the double repression regulatory architecture with OR logic. (F) States-and-weights model for a promoter under the double activation regulatory architecture with OR logic.

probability of RNAP being bound is given by

$$p_{\text{bound}} = \frac{\frac{P}{N_{\text{NS}}} e^{-\beta \Delta\varepsilon_{\text{pd}}}}{1 + \frac{P}{N_{\text{NS}}} e^{-\beta \Delta\varepsilon_{\text{pd}}}}, \tag{55}$$

where N_{NS} is the number of non-specific binding sites; P is the number of RNAP; $\Delta\varepsilon_{\text{pd}}$ is the binding energy of the RNAP.

For a promoter with the simple repression regulatory architecture, the states-and-weights diagram is shown in Fig S3(B), and the probability of RNAP being bound is given by

$$p_{\text{bound}} = \frac{\frac{P}{N_{\text{NS}}} e^{-\beta \Delta\varepsilon_{\text{pd}}}}{1 + \frac{P}{N_{\text{NS}}} e^{-\beta \Delta\varepsilon_{\text{pd}}} + \frac{R}{N_{\text{NS}}} e^{-\beta \Delta\varepsilon_{\text{rd}}}}, \tag{56}$$

where N_{NS} is the number of non-specific binding sites; P is the number of RNAP; R is the number of repressors; $\Delta\varepsilon_{\text{pd}}$ is the binding energy of the RNAP; $\Delta\varepsilon_{\text{rd}}$ is the binding energy of the repressor. Here, the weak promoter approximation is often made, which states that the RNAP binding state has a much lower Boltzmann weight compared to the repressor binding site. Therefore, the expression can often be simplified to

$$p_{\text{bound}} = \frac{\frac{P}{N_{\text{NS}}} e^{-\beta \Delta\varepsilon_{\text{pd}}}}{1 + \frac{R}{N_{\text{NS}}} e^{-\beta \Delta\varepsilon_{\text{rd}}}}. \tag{57}$$

For a promoter with the simple activation regulatory architecture, the states-and-weights diagram is shown in Fig S3(C), and the probability of RNAP being bound is given by

$$p_{\text{bound}} = \frac{\frac{P}{N_{\text{NS}}} e^{-\beta \Delta\varepsilon_{\text{pd}}} + \frac{P}{N_{\text{NS}}}\frac{A}{N_{\text{NS}}} e^{-\beta(\Delta\varepsilon_{\text{pd}} + \Delta\varepsilon_{\text{ad}})}\omega_{\text{ap}}}{1 + \frac{P}{N_{\text{NS}}} e^{-\beta \Delta\varepsilon_{\text{pd}}} + \frac{A}{N_{\text{NS}}} e^{-\beta \Delta\varepsilon_{\text{rd}}} + \frac{P}{N_{\text{NS}}}\frac{A}{N_{\text{NS}}} e^{-\beta(\Delta\varepsilon_{\text{pd}} + \Delta\varepsilon_{\text{ad}})}\omega_{\text{ap}}}, \tag{58}$$

where N_{NS} is the number of non-specific binding sites; P is the number of RNAP; A is the number of activators; $\Delta\varepsilon_{\text{pd}}$ is the binding energy of the RNAP; $\Delta\varepsilon_{\text{ad}}$ is the binding energy of the activator; $\omega_{\text{a}_1\text{a}_2}$ is the interaction energy between the activator and the RNAP.

For a promoter with the repression-activation regulatory architecture, the states-and-weights diagram is shown in Fig S3(D), and the probability of RNAP being bound is given by

$$p_{\text{bound}} = \frac{\frac{P}{N_{\text{NS}}} e^{-\beta \Delta\varepsilon_{\text{pd}}} + \frac{P}{N_{\text{NS}}}\frac{A}{N_{\text{NS}}} e^{-\beta(\Delta\varepsilon_{\text{pd}} + \Delta\varepsilon_{\text{ad}})}\omega_{\text{ap}}}{1 + \frac{P}{N_{\text{NS}}} e^{-\beta \Delta\varepsilon_{\text{pd}}} + \frac{R}{N_{\text{NS}}} e^{-\beta \Delta\varepsilon_{\text{rd}}} + \frac{A}{N_{\text{NS}}} e^{-\beta \Delta\varepsilon_{\text{ad}}} + \frac{P}{N_{\text{NS}}}\frac{A}{N_{\text{NS}}} e^{-\beta(\Delta\varepsilon_{\text{pd}} + \Delta\varepsilon_{\text{ad}})}\omega_{\text{ap}}}, \tag{59}$$

where N_{NS} is the number of non-specific binding sites; P is the number of RNAP; R is the number of repressors; A is the number of activators; $\Delta\varepsilon_{\text{pd}}$ is the binding energy of the RNAP; $\Delta\varepsilon_{\text{rd}}$ is the binding energy of the repressor; $\Delta\varepsilon_{\text{ad}}$ is the binding energy of the activator; $\omega_{\text{a}_1\text{a}_2}$ is the interaction energy between the activator and the RNAP.

Let $r_1 = \frac{R_1}{N_{\text{NS}}} e^{-\beta \Delta\varepsilon_{r_1 d}}$, $r_2 = \frac{R_2}{N_{\text{NS}}} e^{-\beta \Delta\varepsilon_{r_2 d}}$, and $p = \frac{P}{N_{\text{NS}}} e^{-\beta \Delta\varepsilon_{pd}}$. Then, for a promoter with the double repression regulatory architecture under OR logic, the states-and-weights diagram is shown in Fig S3(E), and the probability of RNAP being bound is given by

$$p_{\text{bound}} = \frac{p}{1 + r_1 + r_2 + r_1 r_2 \omega_{\text{r}_1\text{r}_2} + p}, \tag{60}$$

where N_{NS} is the number of non-specific binding sites; P is the number of RNAP; R_1 is the number of the first repressor; R_2 is the number of the second repressor; $\Delta\varepsilon_{\text{pd}}$ is the binding energy of the RNAP; $\Delta\varepsilon_{\text{r}_1\text{d}}$ is the binding energy of the first repressor; $\Delta\varepsilon_{\text{r}_2\text{d}}$ is the binding energy of the second repressor; $\omega_{\text{r}_1\text{r}_2}$ is the interaction energy between the two repressors. The states-and-weights diagram of the AND-logic double repression regulatory architecture is shown in Fig 7(A). In this case, the probability of RNAP being bound is given by

$$p_{\text{bound}} = \frac{p + r_1 p + r_2 p}{1 + r_1 + r_2 + r_1 r_2 \omega_{\text{r}_1\text{r}_2} + p + r_1 p + r_2 p}, \tag{61}$$

Let $a_1 = \frac{A_1}{N_{\text{NS}}} e^{-\beta\Delta\varepsilon_{a_1d}}$, $a_2 = \frac{A_2}{N_{\text{NS}}} e^{-\beta\Delta\varepsilon_{a_2d}}$, and $p = \frac{P}{N_{\text{NS}}} e^{-\beta\Delta\varepsilon_{pd}}$. Then, for a promoter with the double repression regulatory architecture under OR logic, the states-and-weights diagram is shown in Fig S3(F), and the probability of RNAP being bound is given by

$$p_{\text{bound}} = \frac{p + a_1 p\omega_{a_1p} + a_2 p\omega_{a_2p} + a_1a_2p\omega_{a_1p}\omega_{a_2p}}{1 + a_1 + a_2 + p + a_1a_2\omega_{a_1a_2}p + a_1p\omega_{a_1p} + a_2p\omega_{a_2p} + a_1a_2p\omega_{a_1p}\omega_{a_2p}}, \quad (62)$$

where N_{NS} is the number of non-specific binding sites; P is the number of RNAP; A_1 is the number of the first activator; A_2 is the number of the second activator; $\Delta\varepsilon_{\text{pd}}$ is the binding energy of the RNAP; $\Delta\varepsilon_{\text{a}_1\text{d}}$ is the binding energy of the first activator; $\Delta\varepsilon_{\text{a}_2\text{d}}$ is the binding energy of the second activator; $\omega_{\text{a}_1\text{p}}$ is the interaction energy between the first activator and the RNAP; $\omega_{\text{a}_1\text{a}_2}$ is the interaction energy between the second activator and the RNAP. The states-and-weights diagram of the AND-logic double activation regulatory architecture is shown in Fig 8(A). In this case, the probability of RNAP being bound is given by

$$p_{\text{bound}} = \frac{p + a_1 p\omega_{a_1p} + a_2 p\omega_{a_2p} + a_1a_2p\omega_{a_1p}\omega_{a_2p}\omega_{a_1a_2}}{1 + a_1 + a_2 + p + a_1a_2\omega_{a_1a_2}p + a_1p\omega_{a_1p} + a_2p\omega_{a_2p} + a_1a_2p\omega_{a_1p}\omega_{a_2p}\omega_{a_1a_2}}, \quad (63)$$

where $\omega_{\text{a}_1\text{a}_2}$ is the interaction energy between the two activators.

S4 Appendix Recovering binding site signal under extreme mutation rates

As we have shown in Sec 1.2, when the rate of mutation in the mutant library is low, we lose the signal at the RNAP binding site. We hypothesize that this is because RNAP binds weakly at the promoter. We generated a synthetic dataset that has low mutation rate but stronger binding energy at the RNAP binding site. As shown in Fig S4(A), the information footprint built from this dataset has a much higher level of mutual information at the RNAP binding site compared to the information footprint built from a dataset with the same mutation rate but weak RNAP binding energy, which supports our hypothesis.

We also showed that when the rate of mutation in the mutant library is high, there is low mutual information at the repressor binding site. Our hypothesis is that this is caused by the large effects of mutations on the repressor binding energy. We generated a synthetic dataset with high mutation rate while reducing the effect of mutation on binding energy by five fold. As shown in Fig S4(B), this allows us to recover the signal at the repressor binding site, which is also in line with our hypothesis.

S5 Appendix Transcription factor knock-out under double activation

A double-activation promoter can also operate under an AND or an OR logic gate [2]. The states-and-weights diagram for a double-activation promoter is shown in Fig S5(A). Under AND logic, the two activators can interact both with the RNAP and with each other. This cooperativity leads to a further increase in expression levels. In contrast, under OR logic, the activators independently interact with RNAP and there is no cooperativity between them. We build synthetic datasets for an AND-logic and an OR-logic double-activation promoter. As shown in Fig S5(B) and S5(C), under AND logic, since cooperativity is at play, the signal at both A_1 and A_2 binding sites increases when A_1 is increased. On the other hand, under OR logic, the two activators act independently and there is competition between the signals at the two sites. When A_1 is increased, the signal at A_1 binding site correspondingly increases but the signal at A_2 binding site decreases.

S6 Appendix Changing inducer concentration for the inducible activator

In Sec 2.4, we discussed the effects of inducer concentration on the information footprints of a simple repression promoter with an inducible repressor. Similar effects can also be seen for a simple activation

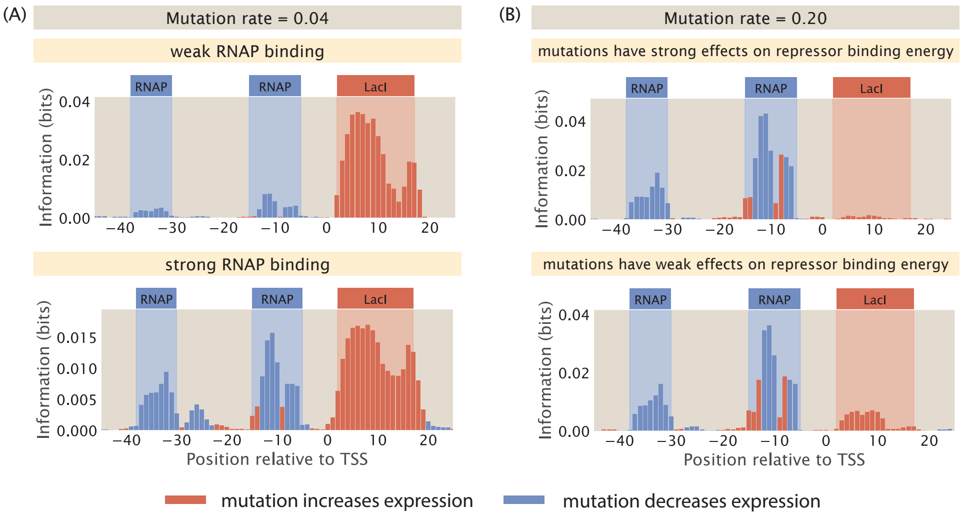

Supplementary Figure 4. Recovering signals from information footprints under extreme mutation rates. (A) We generated two synthetic datasets with a mutation rate of 0.04 in the mutant library. In the first dataset, we set the RNAP binding energy $\Delta\varepsilon_{rd}$ to be $-5\ k_BT$, which is typical of RNAP binding at the wild type -10 and -35 binding sites. In the footprint produced from this dataset, there is low mutual information at the RNAP binding site due to the low mutation rate. On the other hand, in the second dataset, we increased $\Delta\varepsilon_{rd}$ to $-12\ k_BT$. This allows us to recover the signal at the RNAP binding site. (B) We generated two synthetic datasets with a mutation rate of 0.20. In the first dataset, we used the experimentally measured energy matrix for LacI at the O1 operator shown in Fig 2(B), where the average effect of mutations on binding energy is 2.24 k_BT. In the footprint produced from this dataset, there is low mutual information at the repressor binding site due to the high mutation rate. In the second dataset, we reduced the average effect of mutations fivefold and are able to recover the signal at the repressor binding site.

promoter with an inducible activator. One example of an inducible activator is CRP, which changes its conformation when bound to cyclic-AMP and thereby becomes more favorable to DNA binding [3]. Based on the states-and-weights diagram for such a promoter, which is shown in Fig S6(A), the probability of RNAP being bound is given by

$$p_{\text{bound}} = \frac{p + pa_A\omega_A + pa_I\omega_I}{1 + p + a_A + a_I + pa_A\omega_A + pa_I\omega_I}, \tag{64}$$

where p is the normalized weight of the RNAP bound state, a_A is the normalized weight of the active activator bound state, and a_I is the normalized weight of the inactive activator bound state. ω_A and ω_I account for the interaction energy between the RNAP and the active activator and the interaction energy between the RNAP and the inactive activator, respectively. The exact expressions for p, a_A, a_I, ω_A and ω_I are given in Fig S6(A).

To simplify the expression above, we determine the proportion of active and inactive activators with respect to the total number of activators. Similar to the case of simple repression, we calculate $p_{\text{active}}(c)$, which is the probability that the activator exists in the active conformation as a function of the inducer concentration, c. The different states of the activator can be modelled using the states-and-weights diagram shown in Fig S6(B). Here, we consider two types of cooperativity. The first type of cooperativity is between the two binding sites, where each ligand binding event changes the binding affinity of the unbound site. This is inherent to the classic Monod–Wyman–Changeux model and is already encoded in the terms ω_A and ω_I in Eqn. 64. The second type of cooperativity is between the two ligands, which accounts for the negative cooperativity of CRP in the inactive state. This is accounted for by the cooperative energy terms $\varepsilon^A_{\text{int}}$ and $\varepsilon^I_{\text{int}}$, which represent the interaction energies between the two ligands in the active and inactive states,

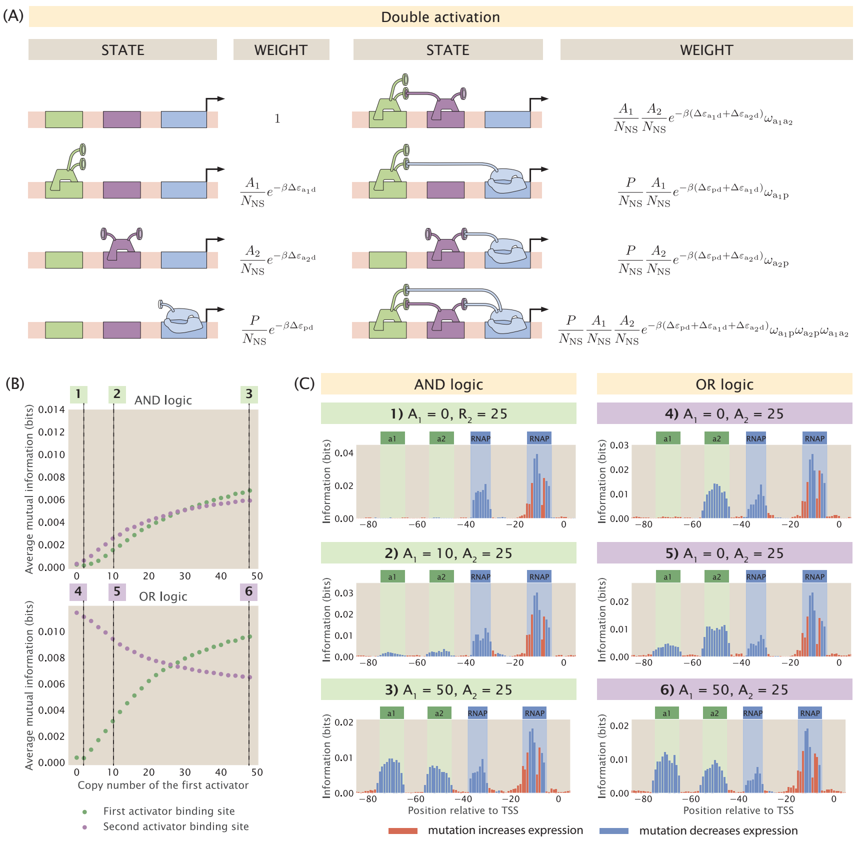

Supplementary Figure 5. Changing the copy number of activators in a double activation promoter. (A) States-and-weights diagram of a promoter with the double activation regulatory architecture. Under OR logic, the two activators do not exhibit coorperativity and $\omega_{\mathrm{a_1 a_2}} = 0\ k_BT$. The states-and-weights diagram of a double activation promoter with OR logic is also shown in Fig S2(F). (B) Changing the copy number of the first activator under AND logic and OR logic affects the signal at both activator binding sites. The energy matrices of the activators are randomly generated in the same way as the energy matrices of the repressors in Fig 8. For the promoter with AND logic, the interaction energies between the activators and between the activator and the RNAP are set to -4 k_BT. For the promoter with OR logic, the interaction energies between the activators and between the activator and the RNAP are set to -7 k_BT. The higher interaction energy for the OR logic promoter is to ensure that there are similar levels of signal at the activator binding sites compared to the AND logic promoter. All data points are the mean of average mutual information across 20 synthetic datasets with the same parameters. (C) Representative information footprints of a double repression promoter under AND and OR logic.

respectively. Given the states-and-weights diagram, $p_{\mathrm{active}}(c)$ is given by

$$p_{\mathrm{active}}(c) = \frac{1 + \frac{c}{K_L^A} + \frac{c}{K_R^A} + \frac{c}{K_L^A}\frac{c}{K_R^A} e^{-\beta \varepsilon_{\mathrm{int}}^A}}{1 + \frac{c}{K_L^A} + \frac{c}{K_R^A} + \frac{c}{K_L^A}\frac{c}{K_R^A} e^{-\beta \varepsilon_{\mathrm{int}}^A} + e^{-2\beta\varepsilon_{AI}} (1 + \frac{c}{K_L^I} + \frac{c}{K_R^I} + \frac{c}{K_L^I}\frac{c}{K_R^I} e^{-\beta \varepsilon_{\mathrm{int}}^I})}, \tag{65}$$

where K_L^A is the dissociation constant between the inducer and the left binding pocket of the active activator, K_R^A is the dissociation constant between the inducer and the right binding pocket of the active activator, K_L^I is the dissociation constant between the inducer and the left binding pocket of the inactive activator, and K_R^I is the dissociation constant between the inducer and the right binding pocket of the inactive activator. With this expression, we can represent the number of active and inactive activators as $A_A = p_{\mathrm{active}} A$ and $A_I = (1 - p_{\mathrm{active}})A$. Therefore, we have that $a_A = p_{\mathrm{active}} \frac{A}{N_{\mathrm{NS}}} e^{-\beta \Delta\varepsilon_{ad}^A}$ and $a_I = (1 - p_{\mathrm{inactive}}) \frac{A}{N_{\mathrm{NS}}} e^{-\beta \Delta\varepsilon_{ad}^I}$.

We built synthetic datasets for a simple activation promoter with an inducible activator. As shown in

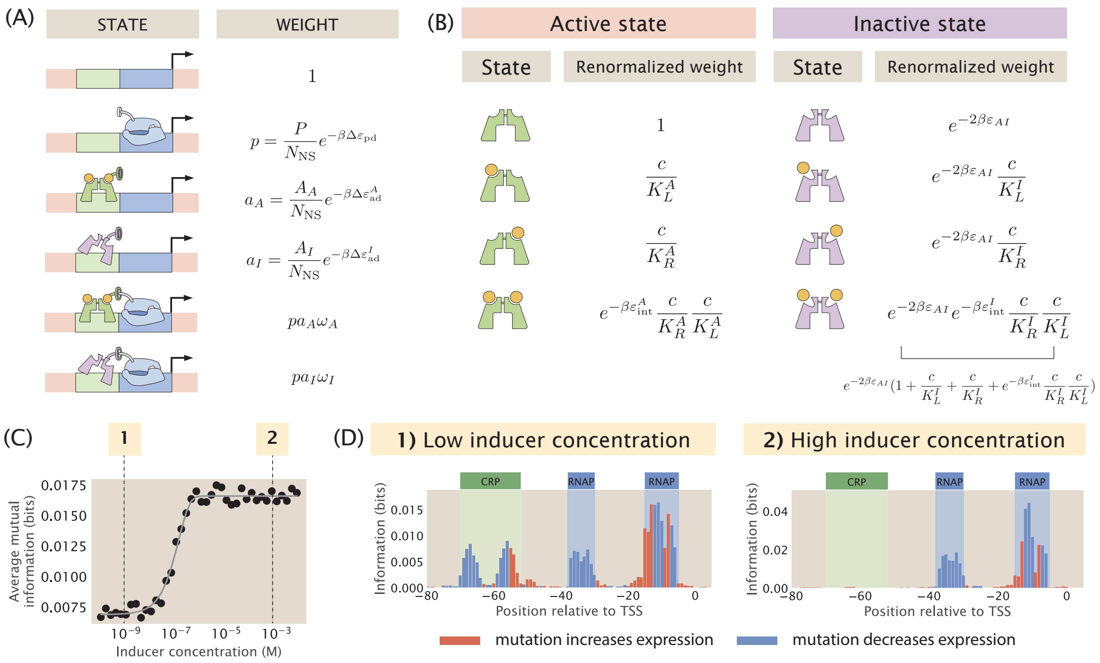

Supplementary Figure 6. Changing inducer concentration for the inducible activator. (A) States-and-weights diagram for an inducible activator. In the diagram, N_{NS} is the number of non-binding sites in the genome, P is the copy number of the RNAP, A_A is the copy number of active activators, A_I is the copy number of inactive activators, $\Delta\varepsilon_{\text{pd}}$ is the binding energy of the RNAP, $\Delta\varepsilon_{\text{ad}}^{A}$ is the binding energy of the active activator, $\Delta\varepsilon_{\text{ad}}^{I}$ is the binding energy of the inactive activator. $\omega_A = e^{-\beta\varepsilon_{p,a_A}}$ and $\omega_I = e^{-\beta\varepsilon_{p,a_I}}$, where ε_{p,a_A} is the interaction energy between the RNAP and the active activator and ε_{p,a_I} is the interaction energy between the RNAP and the inactive activator. (B) States-and-weights diagram to calculate the probability that the activator is in the active state. (C) Average mutual information at the RNAP binding site increases as the inducer concentration increases. Here, we let $K_L^A = K_R^A = 3 \times 10^{-6}$ M, $K_L^I = K_R^A = 10^{-7}$ M, and $\Delta\varepsilon_{AI} = -3\ k_BT$ [3]. (D) Representative information footprints with low inducer concentration (10^{-9} M) and high inducer concentration (10^{-3} M).

Fig S6(C) and Fig S6(D), when the concentration of the inducer is increased, the average signal at the RNAP binding site increases, which corresponds to an increase in expression. At high inducer concentration, the activator is too strongly bound to be affected by mutations, and therefore the signal at the activator binding site is negligible.

S7 Appendix Noise from experimental procedures

In MPRAs such as Reg-Seq, the mutant library is grown up in culture. Once the cell culture is prepared, genomic DNA (gDNA) and mRNAs are extracted, the latter of which is used as a template in reverse transcription to make complementary DNA (cDNA). Afterwards, polymerase chain reation (PCR) is performed to amplify the reporter gene from the gDNA and cDNA. Finally, sequencing adapters are attached to the gDNA and cDNA. The gDNA and cDNA are then sequenced to obtain DNA and RNA counts for each sequence variant.

As illustrated in Fig S7(A), there are at least two possible sources of experimental noise. Firstly, PCR amplification is a stochastic process where the probability that a DNA molecule is amplified in a given cycle is less than one. This stochasticity may cause some sequences to have an artificially high RNA count. We note that assuming that the same reporter gene is used for each sample, the only difference in the sequence being amplified would be the barcode. Since barcodes are typically much shorter, it is unlikely to signficiantly alter the GC-content of the sequence, and therefore we do not discuss the effect of PCR sequence bias.

Secondly, during RNA-Seq as well as the prior library preparation procedures such as RNA extraction and reverse transcription, we cannot ensure that every mRNA is extracted, converted to cDNA, and sequenced. Instead, in these steps, only a random subset of the original pool of mRNAs is sampled and included in the final sequencing dataset. As a result, a sequence may have an artificially low RNA count because some copies of the mRNA associated with that sequence are not sampled in one of the experimental steps.

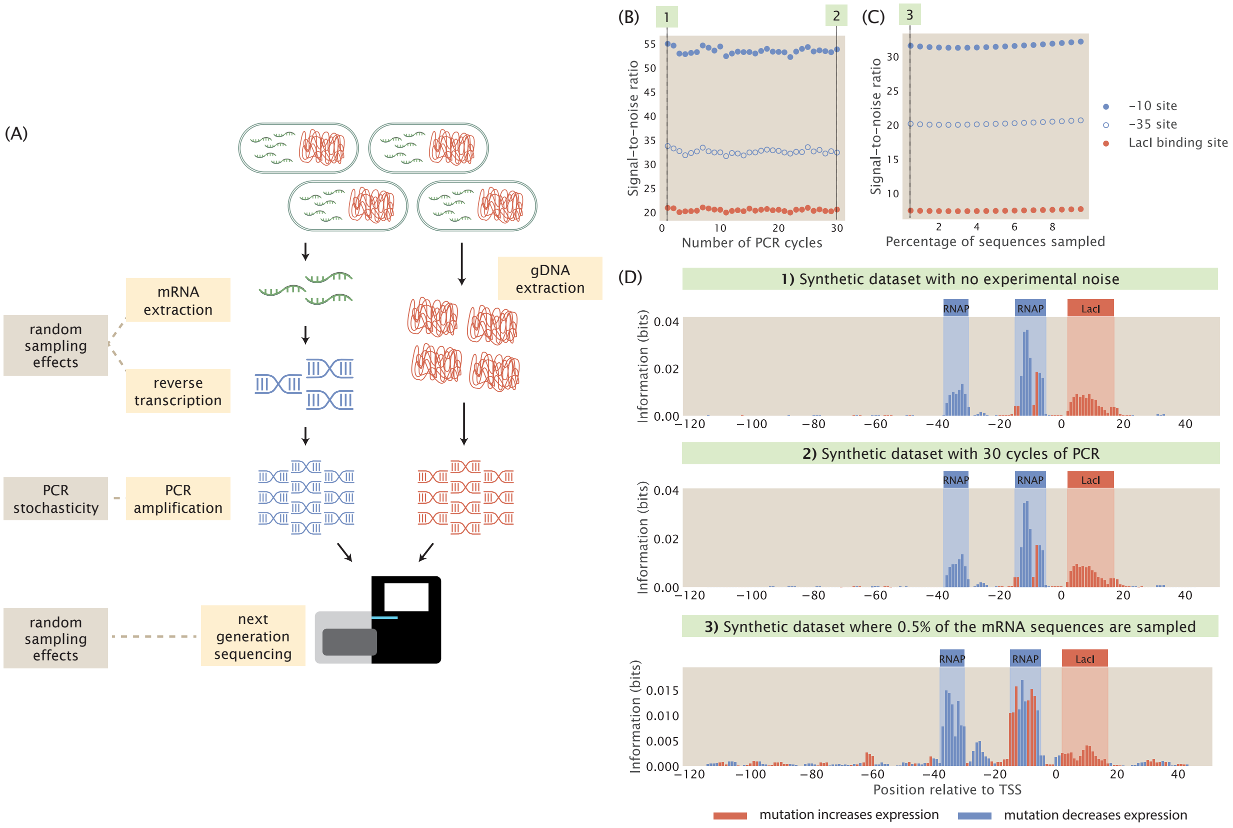

Supplementary Figure 7. Noise from experimental procedures in the Reg-Seq pipeline. (A) The two main sources of noise in the experimental MPRA pipeline are stochasticity from PCR amplification and random sampling effects from RNA extraction, reverse transcription, and RNA-Seq. (B) Signal-to-noise ratio in the information footprints remains high when the number of PCR amplification cycles is increased. Here, $P_{\text{amp}} = 0.5$. (C) Signal-to-noise ratio remains high when only a small percentage of the sequences are randomly sampled. (D) Representative information footprints with no experimental noise, PCR stochasticity after 30 cycles, and random sampling effects after 0.5% of the RNA sequences are sampled.

We simulate these two sources of experimental noise in our computational pipeline. To simulate PCR with n cycles of amplification, we start with the original mRNA counts predicted based on the probability of RNAP being bound. Subsequently, we model the number of sequences that are successfully amplified during each cycle using a Binomial distribution [4]. Hence, for each sequence variant,

$$n(j+1) = n(j) + B(n(j), P_{\text{amp}}), \tag{66}$$

where $n(j)$ is the number of sequences of the promoter variant in cycle j, $B(n, P)$ models the Binomial distribution, and P_{amp} is the probability that a sequence is successfully amplified in a cycle. We applied Eqn 66 to calculate the final count of each sequence variant in a library. As shown in Fig S7(B) and S7(D), even when the probability of amplification is set to a low number of $P_{\text{amp}} = 0.5$, increasing the number of PCR cycles does not reduce the signal-to-noise ratio in information footprints. Therefore, we conclude that stochasticity in PCR does not contribute to significant levels of noise in information footprints.

To simulate the random sampling effect during RNA extraction, reverse transcription, and sequencing, we randomly draw a subset of promoter variants in the mutant library and we only consider the expression levels

of the selected promoter variants when we calculate mutual information to build the information footprint. As shown in Fig S7(C) and S7(D), the levels of noise only becomes significant when less than 1% of the original pool of sequences is sampled. Therefore, random sampling effects are not a significant source of noise in information footprints either.

S8 Appendix Modelling extrinsic noise using a log-normal distribution

In order to account for extrinsic noise, we choose to use a log-normal distribution to describe the copy number of RNAPs and repressors. To model the copy number of RNAPs, the parameters of the underlying Normal distribution are

$$\mu_{\text{RNAP}} = \log P \tag{67}$$

$$\sigma_{\text{RNAP}} = \alpha_{\text{RNAP}} \cdot \mu_{\text{RNAP}}, \tag{68}$$

where $P = 5000$ is the reporter copy number of RNAPs in *E. coli.* The magnitude of σ_{RNAP} is defined as a product between μ_{RNAP} and the coefficient of variation α_{RNAP}. We draw a random number β_{RNAP} from this Normal distribution and the final copy number of RNAP is given by

$$P_{\text{with noise}} = e^{\beta_{\text{RNAP}}}. \tag{69}$$

Similarly, to model the copy number of the repressors, we use another Normal distribution with the parameters

$$\mu_{\text{repressor}} = \log R \tag{70}$$

$$\sigma_{\text{repressor}} = \alpha_{\text{repressor}} \cdot \mu_{\text{repressor}}, \tag{71}$$

where $R = 10$ is the reporter copy number of LacI in *E. coli* and $\alpha_{\text{repressor}}$ is the coefficient of variation. We draw a random number $\beta_{\text{repressor}}$ from this Normal distribution and the final copy number of the repressors is given by

$$R_{\text{with noise}} = e^{\beta_{\text{repressor}}}. \tag{72}$$

We increase $\alpha_{\text{RNAP}} = \alpha_{\text{repressor}}$, which increases the standard deviation in the underlying Normal distributions and therefore the extrinsic noise in the datasets. The distribution of RNAP and repressor copy numbers using low and high values of $\alpha_{\text{RNAP}} = \alpha_{\text{repressor}}$ is shown in Fig S8.

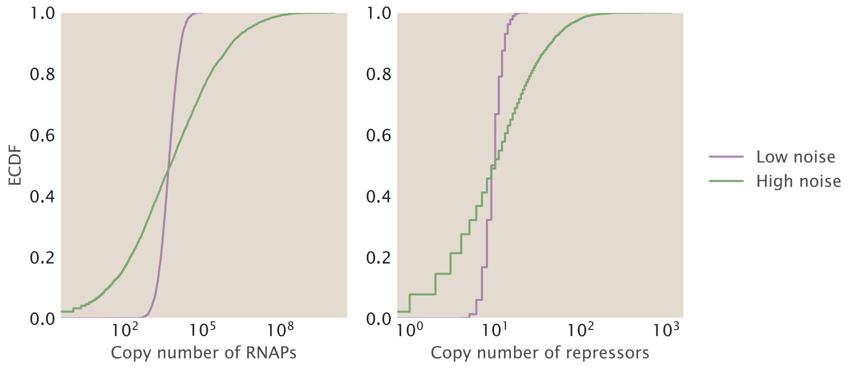

Supplementary Figure 8. Modelling the copy number of RNAPs and repressors using a log-normal distribution. Empirical CDFs for the copy number of RNAPs and repressors modelled using a log-normal distribution. For the ECDFs with low extrinsic noise, $\alpha_{\text{RNAP}} = \alpha_{\text{repressor}} = 0.1$. For the ECDFs with high extrinsic noise, $\alpha_{\text{RNAP}} = \alpha_{\text{repressor}} = 0.5$.

SI references

1. Bintu L, Buchler NE, Garcia HG, Gerland U, Hwa T, Kondev J, et al. Transcriptional regulation by the numbers: models. Curr Opin Genet Dev. 2005;15(2):116–124.

2. Buchler NE, Gerland U, Hwa T. On schemes of combinatorial transcription logic. Proc Natl Acad Sci U S A. 2003;100(9):5136–5141.

3. Einav T, Duque J, Phillips R. Theoretical analysis of inducer and operator binding for cyclic-AMP receptor protein mutants. PLoS One. 2018;13(9):e0204275.

4. Kebschull JM, Zador AM. Sources of PCR-induced distortions in high-throughput sequencing data sets. Nucleic Acids Res. 2015;43(21):e143.
